# Quality by Design Approach Using Multiple Linear and Logistic Regression Modeling Enables Microemulsion Scale Up

**DOI:** 10.3390/molecules24112066

**Published:** 2019-05-30

**Authors:** Michele Herneisey, Eric Lambert, Allison Kachel, Emma Shychuck, James K. Drennen, Jelena M. Janjic

**Affiliations:** 1 Graduate School of Pharmaceutical Sciences, Duquesne University, Pittsburgh, PA 15228, USA; herneiseym@duq.edu (M.H.); lamberte@duq.edu (E.L.); kachela@duq.edu (A.K.); shychucke@duq.edu (E.S.); drennen@duq.edu (J.K.D.III); 2 Chronic Pain Research Consortium, Duquesne University, Pittsburgh, PA 15228, USA

**Keywords:** quality by design (QbD), modeling, microemulsions

## Abstract

The development of pharmaceutical nanoformulations has accelerated over the past decade. However, the nano-sized drug carriers continue to meet substantial regulatory and clinical translation challenges. In order to address some of these key challenges in early development, we adopted a quality by design approach to develop robust predictive mathematical models for microemulsion formulation, manufacturing, and scale-up. The presented approach combined risk management, design of experiments, multiple linear regression (MLR), and logistic regression to identify a design space in which microemulsion colloidal properties were dependent solely upon microemulsion composition, thus facilitating scale-up operations. Developed MLR models predicted microemulsion diameter, polydispersity index (PDI), and diameter change over 30 days storage, while logistic regression models predicted the probability of a microemulsion passing quality control testing. A stable microemulsion formulation was identified and successfully scaled up tenfold to 1L without impacting droplet diameter, PDI, or stability.

## 1. Introduction

The publication rate in drug delivery using nanoformulations has dramatically increased in the past decade, reaching 24,665 publications by 2017 [1]. However, this rapid increase in publications has coincided with a limited number of clinical trials and approved treatments. Several explanations have been proposed to justify the lack of clinical translation of nanoformulations for drug delivery, including a lack of translatability from animal models to humans, an overemphasis on the advantages of nanoformulations, confirmation bias, and the development of increasingly complex nanoformulations [1]. These problems are all relevant and need to be addressed in order for nanoformulations to reach the market. We postulate that quality by design approaches used throughout the pharmaceutical industry could be adapted to nanoformulation development. Doing so may allow challenges to be identified and corrected early on, resulting in higher quality nanoformulations during early developmental stages.

Quality by design (QbD) is defined by the International Council for Harmonization (ICH) Q8(R2) document as “a systematic approach to development that begins with predefined objectives and emphasizes product and process understanding and process control, based on sound science and quality risk management”. A QbD approach includes but is not limited to (1) identification of critical quality attributes (CQAs), (2) risk analysis, (3) design of experiments (DoE), and (4) identification of critical process parameters (CPPs). CQAs are measurable quality attributes that have a significant impact on final product quality. After CQA identification, risk analyses such as a failure mode, effects, and criticality analysis (FMECA) are used to strategically rank potential failure modes and narrow down the list of process parameters to those that are most likely to significantly affect the CQAs [2]. DoE is then developed to study the relationships between the process parameters and the CQAs in an efficient way. DoE allows for sophisticated studies in which multiple factors are changed at a time, minimizing the required number of experimental runs and increasing efficiency compared to traditional one factor at a time approaches [3]. CQAs are measured for each run of the DoE, and statistical regression approaches (multiple linear regression, logistic regression, etc.) or other modeling approaches are used to identify the CPPs, the process parameters that have a significant impact on the CQAs. 

QbD approaches can lead to the development of quality nanoformulations while minimizing risk, time, and resources. QbD has been applied to a wide variety of nano-sized formulations in recent years, including liposomes, emulsions, particles, micelles, and suspensions [4]. Recent examples include liposomes co-encapsulating doxorubicin and curcumin [5], topical microemulsion-based hydrogels containing itraconazole [6], aceclofenac-loaded nanostructured lipid carriers [7], rosuvastatin calcium solid lipid nanoparticles [8], quercetin-salicylic acid nanomixed micelles [9], and furosemide nanosuspensions [10]. Presented here, microemulsions are used as a model to demonstrate the usefulness and application of QbD approaches to nanoformulation development. Microemulsions are optically transparent emulsions that typically range from 20–100 nm in diameter [11]. Microemulsions are thermodynamically stable, allowing them to undergo emulsification spontaneously, a process known as emulsion inversion. During emulsion inversion, the emulsion proceeds through an intermediate phase of minimal surface tension to invert from a water in oil to an oil in water emulsion, or vice versa [12]. Emulsification can be directed through modification of the system’s salinity, pH, or temperature, all of which can modify the hydrophilic/lipophilic balance [13]. Alternatively, emulsification can occur through composition modification (e.g. modification of water to oil ratio), a process known as the water titration method [12]. Microemulsion formulation via water titration is common, and many groups have used design of experiments or QbD to develop microemulsions using water titration. Many of these reports focus solely on microemulsion composition. However, several reports have found that the process parameters water addition rate and stir rate impact microemulsion diameter [14,15,16]. To increase the chances of successful scale up, it is critical to understand the impact, if any, of process parameters on microemulsion properties, such as diameter. Therefore, we propose that a thorough understanding of both composition and process parameters is necessary for quality microemulsion development. Approaches that study both composition and process are known as mixture process variable studies. Mixture process variable approaches have been utilized in the development of microemulsion electrokinetic chromatography methods for the identification of diclofenac [17] and almotriptan [18] and their impurities. To the best of our knowledge, the mixture process variable approach for the development of a microemulsion via water titration has not been reported. 

As mentioned previously, the QbD approach utilizes a statistical experimental design. Statistical regression approaches are used to identify CPPs and describe the relationships between CPPs and CQAs. A variety of modeling methods have been applied to microemulsions, including multiple linear regression (MLR) [19], partial least squares [20,21], logistic regression [22], and artificial neural networks [23,24,25,26]. Artificial neural networks are advantageous, in that they can detect nonlinear relationships between variables and all possible interactions between variables [27]. However, artificial neural networks cannot explicitly approximate the significance of each input variable on the output [27]. Conversely, MLR assigns a numerical value (regression coefficient) to each input variable and evaluates the significance of each input variable on the output. We therefore used MLR to evaluate the impact of microemulsion composition and water titration parameters on day 1 diameter, day 1 polydispersity index (PDI), and 30-day percent diameter increase. A unique aspect of partial least squares is that it can handle more input variables than runs [28], and a unique aspect of logistic regression is that the output variables are binary. Therefore, logistic regression can be used to model a binary response, such as whether a microemulsion formulation meets or fails to meet a CQA specification. For this reason, logistic regression was used to model the probability of microemulsion formulations meeting CQA specifications.

We postulate that nanoformulation quality and process understanding can be improved through the utilization of QbD. In the presented work, we applied QbD methodology to a microemulsion as an example of nanoformulation. The aims of the presented work were (1) to identify stable, robust microemulsions and (2) to understand the processes that impact microemulsion diameter, PDI, and stability. To achieve these aims, we identified CQAs that would facilitate identification of stable microemulsions. We then used FMECA as an example risk analysis approach to identify potential CPPs. These potential CPPs were used to develop a screening mixture process variable DoE. The screening DoE enabled us to identify a design space in which microemulsion diameter, PDI, and stability were dependent solely upon microemulsion composition. We augmented the screening DoE to further explore this design space using MLR and logistic regression models. Specifically, we used MLR to predict microemulsion diameter, PDI, and 30-day percent diameter change as a function of microemulsion composition, and we used logistic regression to evaluate CQAs representative of stress stability. Extensive quality control (thermal cycling and shelf life studies) enabled the development of more accurate logistic models. Improved understanding of these processes allowed us to identify stable, robust microemulsions and successfully scale up a microemulsion formulation tenfold. The marriage of DoE, multiple linear regression, and logistic regression can be used to collect and analyze process information in an efficient manner. This work serves as a demonstration of how QbD approaches can be applied to other classes of nanoformulations. 

## 2. Results

### 2.1. Identification of Critical Quality Attributes (CQAs)

The aims of the presented work were (1) to identify stable, robust microemulsions and (2) to understand the processes that impact microemulsion diameter, PDI, and stability. The selected CQAs reflect this. Microemulsions underwent rigorous evaluation to simulate the stresses experienced upon sterilization, evaluation in in vitro pharmacological studies, transportation, and storage. These evaluative quality control tests included: (1) filtration through a 0.22 μm syringe filter; (2) centrifugation at 1620xg for 30 minutes; (3) thermal cycling test; (4) storage at ambient temperature for 30 days. CQAs were defined as microemulsion diameter change and PDI in response to each of these quality control tests and are listed in Table 1. 

### 2.2. Selection of Microemulsion Excipients

Microemulsions consisted of oil, surfactant, co-surfactant, two solubilizers (transcutol and propylene glycol), and water. Microemulsion excipients, all on the Food and Drug Administration generally regarded as safe list, were selected, such that the final formulation could be adjusted to incorporate different drugs or multiple drugs, depending upon the disease target. Miglyol 812N was chosen as the oil, because it solubilizes lipophilic compounds better than many hydrocarbon oils [29]. Kolliphor EL and transcutol were chosen as the surfactant and primary solubilizer, respectively, because they are the second and third most commonly used excipients in microemulsion formulations [30]. PEG 400 was chosen as the co-surfactant, because multi-drug delivery microemulsion formulations have been developed with a surfactant/co-surfactant combination of Kolliphor EL and PEG 400 in a 1:1 *w*/*w* ratio [31,32]. Finally, propylene glycol was chosen as the second solubilizer, because it is a commonly used solubilizer [30] that is compatible with transcutol. 

### 2.3. Risk Assessment and Selection of Critical Process Parameters

In practice, quality risk assessment considers each unit operation in the manufacture of a pharmaceutical product. In the case of emulsification by water titration, there is one primary unit operation. The water titration method has many variables to consider, including water addition rate, stir rate, vessel size and geometry, temperature, composition, and batch size. Given material and time limitations, it is important to select the most valuable factors to study. In the presented work, failure mode, effects, and criticality analysis (FMECA) was used as a risk assessment tool to strategically rank methods of failure during microemulsion production and identify potential critical process parameters. As part of this risk assessment, a risk priority number was assigned to each failure mode. Risk priority number is calculated as the product of three factors:

(1) RPN=Severity×Frequency of Occurence×Detectability

Severity, frequency of occurrence, and detectability are each represented by a spectrum that rates each factor from 1 to 5 (low to high risk), as shown in Table 2. Potential failure methods were defined for each CQA defined in Table 1, and the process parameters that received the highest risk priority number were identified as potential critical process parameters. FMECA was chosen as the risk analysis method for this work due to its wide applicability in design and manufacturing, its strength in assessment of individual failure modes, and its common use in pharmaceutical manufacturing [33]. An abridged version of the risk assessment is shown in Table 3, and the full risk assessment is shown in the Supplemental Information. FMECA was used in the development of solid self-nanoemulsifying oily formulations (S-SNEOFs) to identify S-SNEOF lipid, surfactant, and co-solubilizer concentrations as high-risk factors [34]. Other groups found that water addition rate and stir rate during water titration impact emulsion diameter [14,15,16], though FMECA was not utilized in these reports. Given this, we postulated that microemulsion composition and water titration process parameters could significantly impact the defined CQAs. These points are reflected in the FMECA (Table 3). The highest risk priority numbers correspond to high oil to surfactant ratio, fast water addition rate, and slow stir rate. To the best of our knowledge, this is the first example of FMECA applied to a microemulsion formulation development. 

### 2.4. Design of Experiments–Screening Design 

Based upon the FMECA, a 15-run D-optimal screening design of experiments was developed to study the impact of stir rate, water addition rate, and microemulsion composition on microemulsion CQAs (runs 1-15, Table 4). CQAs were measured for each run, and the results are presented in Table 5. To assess microemulsion reproducibility, four runs from the screening DoE (2, 3, 8, and 13) were replicated in triplicate, and CQAs were measured for each replicated microemulsion. The average and standard deviation of each triplicated CQA measurement is reported in Table 6. Microemulsions were consistently reproduced and performed similarly under all quality control tests. 

MLR was used to identify the process parameters that were most likely to significantly impact the CQAs of (1) day 1 diameter, (2) day 1 PDI, and (3) 30-day percent diameter change. All process parameters were included in these screening MLR models, and *p*-values for parameter estimates are shown in Table 7. Oil and transcutol significantly contribute to day 1 diameter, while oil, transcutol, and water significantly contribute to day 1 PDI (*p*-value < 0.05). Oil content was the only significant parameter in the 30-day percent diameter change model (*p*-value = 0.0008). Interestingly, water addition rate and stir rate did not have significant *p*-values in any of the MLR models. We concluded that in this specific design space, microemulsion diameter, PDI, and 30-day percent diameter change are dependent solely upon microemulsion composition. Specifically, microemulsion oil content appears to be the most significant predictor of the three studied CQAs, as oil had the lowest p-value in all screening MLR models. 

Two microemulsions (runs 5 and 10) from the screening design failed to meet the day 1 diameter and PDI specifications (Table 5). Specifically, day 1 diameters were 116.50 and 66.49 nm for runs 5 and 10, respectively. The day 1 diameter for run 5 was found to be a statistical outlier (Grubbs’ test, *p*-value 0.01). Interestingly, runs 5 and 10 had the same composition, but different stir rates and water addition rates. This is inconsistent with the MLR results, which suggest that these parameters do not significantly impact microemulsion diameter. Upon closer inspection, three additional microemulsion pairs had identical composition but a different stir rate or water addition rate (runs 6 and 11, runs 9 and 14, and runs 4 and 15, Table 4). All three pairs had comparable day 1 diameters (less than 1 nm difference, Table 5), which is consistent with the MLR results. We hypothesized that runs 5 and 10 behave differently because they have a higher internal phase (oil, transcutol, and propylene glycol) to surfactants ratio than any other formulation. The internal phase to surfactants ratio was equal to 0.6 for runs 5 and 10, but 0.51 or less for all other formulations. We hypothesized that there is a specific design space in which stir rate and water addition rate do not significantly impact microemulsion diameter and other CQAs, and that a high internal phase to surfactants ratio causes runs 5 and 10 to fall out of this design space. Therefore, runs 5 and 10 were removed from all further analysis.

### 2.5. Design of Experiments–Augmented Design

Through the screening design of experiments, we were able to (1) identify a subset of parameters that significantly impacted microemulsion diameter, PDI, and 30-day percent diameter change; and (2) identify a narrower design space that enabled production of microemulsions in the desired diameter and PDI range. We used this information to add an additional 15 experimental runs to the design of experiments. In this augmented design, the internal phase was adjusted from its original maximum of 13.5% formulation weight to 12%, thus reducing the maximum internal phase to surfactant ratio. Further, the process parameters of water addition rate and stir rate were eliminated from the augmented design. Limiting the number of studied parameters increased the number of levels for each parameter and allowed us to study the impact of interaction terms. Including potential interaction terms has the potential to improve the predictive capabilities of the model. The 15 additional runs added to the DoE are shown in Table 4 (runs 16–30). All microemulsions in the augmented DoE underwent the same quality control tests as the formulations in the screening DoE. The results of these quality control tests are shown in Table 5. 

**Figure 1:**
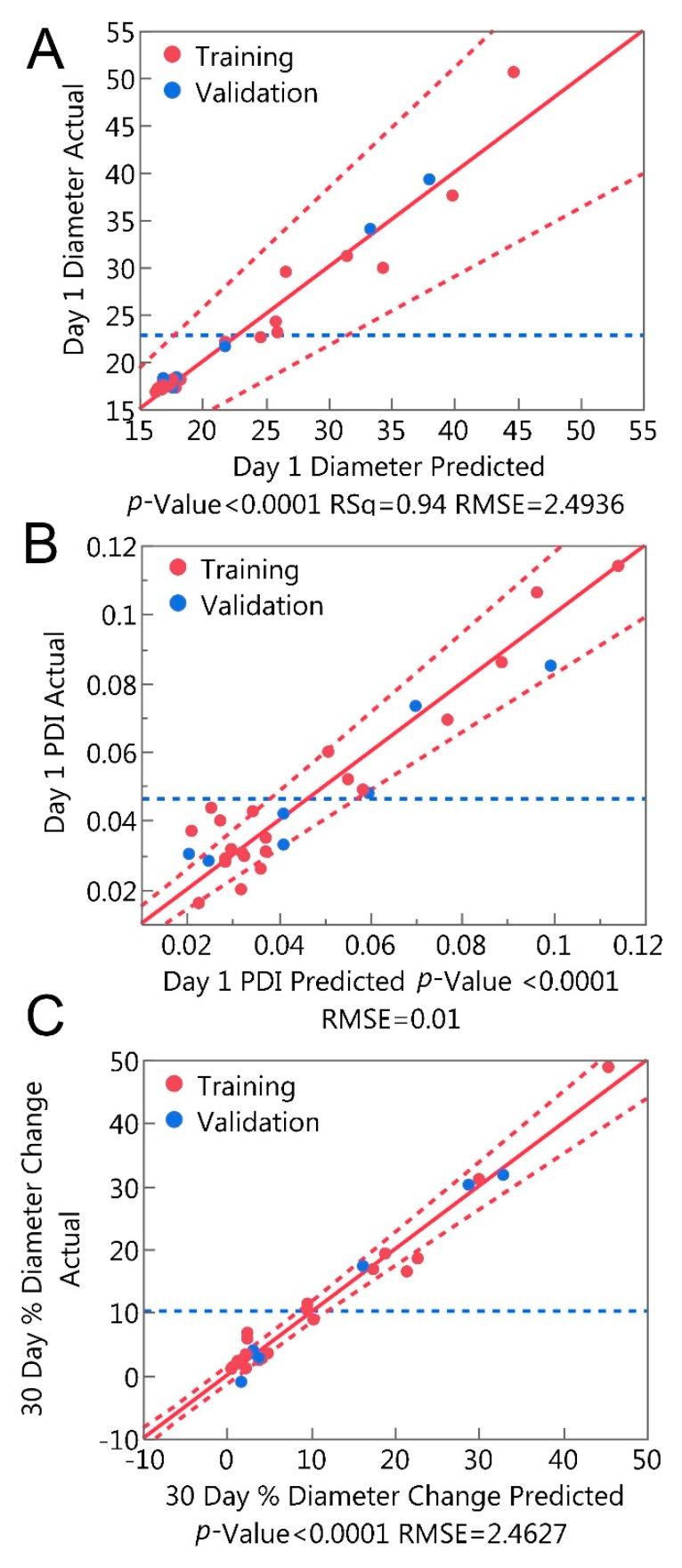
Multiple linear regression models predict microemulsion (**A**) Day 1 diameter; (**B**) Day 1 PDI; (**C**) 30 day % diameter change as a function of microemulsion composition.

Results from the augmented design of experiments were used to develop MLR models for day 1 diameter, day 1 PDI, and 30-day percent diameter change as a function of microemulsion composition. Figure 1 shows a comparison of actual and predicted plots for each model, and model *R*^2^ and RASE values are shown for training and validation sets in Table 8. Model terms, parameter estimates, standard errors, and p-values are shown in Table 9. 

### 2.6. Predicting Microemulsion Stability with Logistic Regression

The majority of microemulsions presented here met the CQA specifications for filtration and centrifugation. However, fourteen formulations failed to meet thermal cycling or 30-day CQA specifications. We therefore chose to focus on the development of logistic regression models that could predict the probability that a microemulsion would meet the CQA specifications for thermal cycling and 30-day stability. 

**Figure 2:**
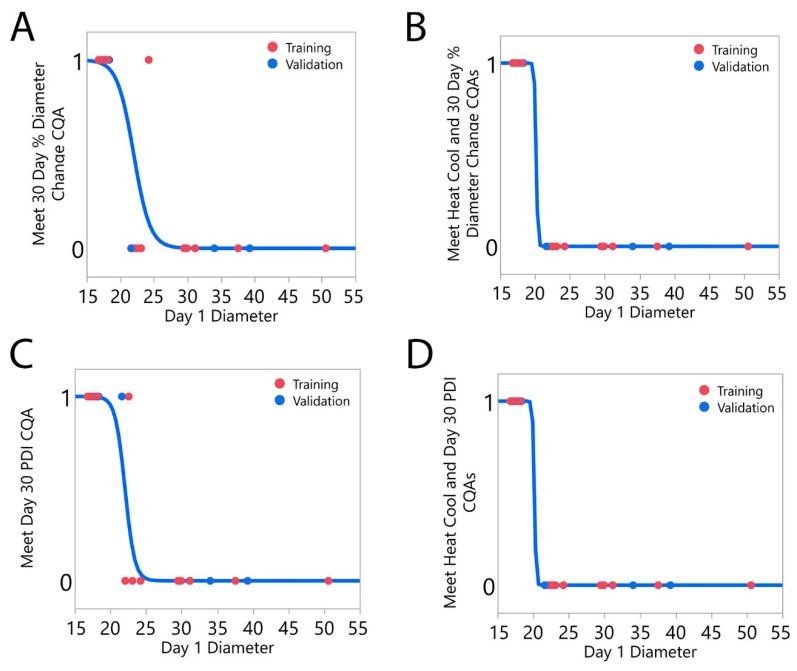
Logistic regression models predict the probability that a microemulsion will meet one or more CQA specifications-based upon its day 1 diameter measurement. (**A**) 30-day percent diameter change; (**B**) 30-day percent diameter change and thermal cycling percent diameter change; (**C**) day 30 PDI; (**D**) day 30 PDI and thermal cycling PDI. The predictive accuracy of the logistic models improves when two CQA specifications must be met.

Upon closer inspection of the data, there appeared to be a trend between day 1 diameter and whether the microemulsion met all CQA specifications. Logistic regression models were developed to predict the probability of a microemulsion meeting the CQA specifications of 30-day percent diameter change (Figure 2A) and day 30 PDI (Figure 2C) as a function of day 1 diameter. The left-hand confusion tables (Table 10) demonstrate the accuracy of these models. The 30-day percent diameter change logistic model has a single misclassification in both the training and validation data sets, and the day 30 PDI logistic model has a misclassification in the training data set. To improve predictive accuracy, logistic models were modified to predict the probability of a microemulsion meeting both the thermal cycling and 30-day CQA specifications. The first of these logistic models predicted whether a microemulsion would meet the 30-day percent diameter change and the thermal cycling diameter change CQA specifications (Figure 2B). Similarly, a logistic model was developed to predict the probability that a microemulsion would meet the day 30 PDI and the thermal cycling PDI CQA specifications (Figure 2D). Modifying the response to incorporate two CQA specifications from different stability tests resulted in more accurate logistic models, as both developed models had zero misclassifications in the training and validation data sets (Table 10). 

### 2.7. Microemulsion Scale-Up to 1 L

**Figure 3:**
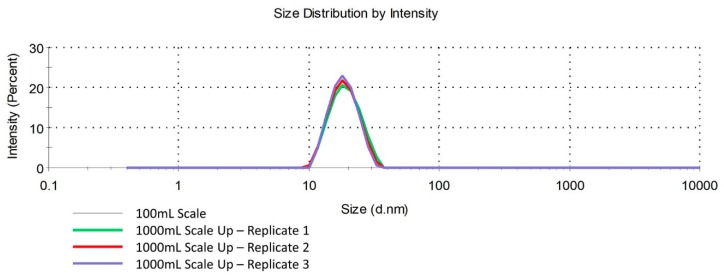
Size distribution by intensity of microemulsions produced on a 1L scale compared to that of a microemulsion produced on a 100mL scale. Scale up of the microemulsion to 1 L can be done consistently and does not impact microemulsion diameter or PDI, suggesting a robust formulation.

Microemulsions were reproduced consistently on a 100 mL scale (Table 6). Further, we found that for this specific design space, water addition rate, and stir rate did not significantly impact microemulsion diameter or PDI. We hypothesized that this would make the microemulsion formulation robust and therefore easier to scale up. We selected formulation 6 (Table 4), because this formulation met all CQA specifications, had a low concentration of surfactants, and had high concentrations of both solubilizers. Therefore, formulation 6 has the potential to solubilize higher concentrations of one or more lipid-soluble drugs. Formulation 6 was scaled up to 1000 mL in triplicate. All scaled up batches underwent the same CQA testing as the 100 mL batches. Microemulsion scale-up was reproducible and comparable to the 100 mL batch (Figure 3, Table 11).

## 3. Discussion

To fully evaluate stress stability of a product, it is critical to have adequate quality control analyses in place. However, there is a lack of extensive, orthogonal quality control analyses in early microemulsion development. Orthogonal quality control analyses such as centrifugation and thermal cycling are becoming more commonly used in microemulsion literature reports [35,36], but this use is inconsistent. Many microemulsion literature reports only investigate droplet diameter and PDI over the span of several months, and microemulsions exhibiting little change in these properties are deemed stable. The time it takes to identify unstable formulations using this approach is prohibitive. Additionally, this approach is not representative of the stresses that microemulsions endure upon transportation or evaluation in in vitro pharmacological studies. In the presented work, we demonstrated that logistic regression could be used to predict the probability that a microemulsion formulation would meet one or more CQA specifications. We also demonstrated that model prediction accuracy was improved when multiple CQA specifications needed to be met simultaneously. This highlights the importance of rigorous quality control analyses early in the development of microemulsions and other nanoformulations. For example, consider run 28, Table 5. This formulation met the CQA specifications for 30-day stability but failed to meet the CQA specifications for thermal cycling. Conversely, runs 13, 16, and 24 (Table 5) met the CQA specifications for thermal cycling but failed to meet the CQA specifications for 30-day stability. Had these formulations been subjected to only one stability test, they may have been deemed stable and proceeded to further testing. The extensive quality control analyses presented here demonstrate that thorough quality control testing early in the development process can aid in the identification of unsuitable or unstable formulations early, saving time and resources. 

**Figure 4:**
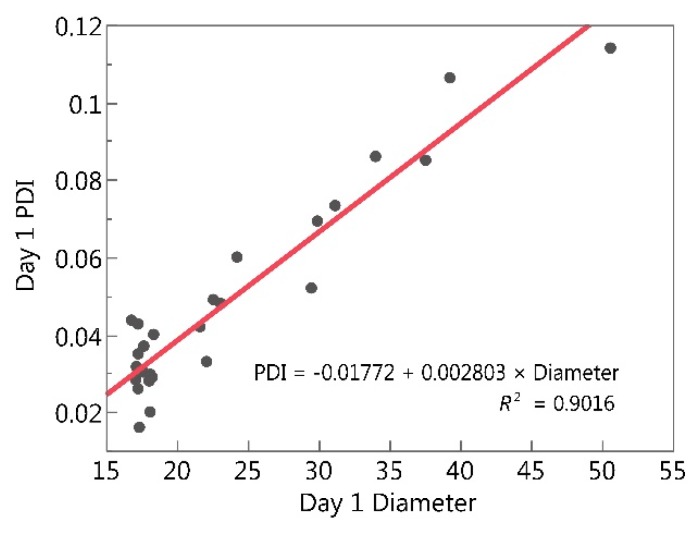
Single linear regression of day 1 microemulsion PDI as a function of day 1 microemulsion diameter.

Interestingly, the MLR models for day 1 diameter and day 1 PDI were similar. The only difference between these two models was that the diameter model included propylene glycol, while this main effect was not significant in the PDI model. It was also interesting that day 1 diameter was able to accurately predict the probability that the CQA specifications for thermal cycling PDI and day 30 PDI were met (Figure 2C,D). To further investigate this potential relationship between diameter and PDI, day 1 PDI was plotted as a function of day 1 diameter, and a single regression line was fit to the data (Figure 4). There is a significant correlation between microemulsion diameter and PDI (*R*^2^ = 0.9016, *p*-value < 0.0001). This is not surprising when considering the methods used to calculate these parameters. Diameter measurements reported here are calculated with zetasizer nano software (Malvern, UK) from the signal intensity, using a cumulants analysis that is the fit of a polynomial to the log of the G1 correlation function [37]. The fitted polynomial is shown in the equation below:

(2) $$\mathrm{Ln}\left[G1\right]=a+bt+ct^{2}$$

The terms *a*, *b*, and *c* are the coefficients of the fitted polynomial, and *t* is time. The second order term is used to define the degree to which the data bear a resemblance to single decay, and the first order term defines diffusion rate, where the coefficient *b* is the z-average diffusion coefficient, used to calculate particle diameter. This coefficient *b*, along with the coefficient *c*, are used to calculate PDI as shown in the below equation:

(3) $$\mathrm{PDI}=\frac{2c}{b^{2}}$$

Since both diameter and PDI are dependent upon the z-average diffusion coefficient *b*, it is reasonable that a correlation would exist between diameter and PDI. The fact that both MLR and logistic regression models confirm this correlation is encouraging and suggests that these statistical regression approaches were able to facilitate our understanding of the relationships between microemulsion composition, diameter, and PDI. 

Models capable of predicting microemulsion stability, including the 30-day percent diameter change MLR model (Figure 1C, Table 8 and Table 9) and the logistic regression models (Figure 2, Table 10) are particularly useful, because they enable the prediction of microemulsion stability prior to production (MLR) or only 24 h after production (logistic regression). This has the potential to save significant time and resources, as microemulsions unlikely to meet CQA specifications can be discarded without running further tests.

Through a screening DoE followed by subsequent augmentation to the DoE, we were able to identify a design space in which stir rate and water addition rate did not have a significant impact on microemulsion diameter, PDI, or stability. This suggests that the presented microemulsion production approach can tolerate changes in the manufacturing process parameters, as long as composition is unchanged. This was confirmed, as we were able to consistently scale up a select microemulsion formulation to ten times its original volume. Successful and reproducible scale up has the potential to ease the transition into extensive animal work or pre-clinical trials, and is therefore useful for nanoformulations in particular, as scale up can prove challenging for these types of formulations. 

Risk assessment using FMECA, in combination with a screening DoE led to the rapid and efficient identification of several stable microemulsion formulations and the identification of a robust design space that enabled microemulsion scale up. Risk analysis narrowed down the number of process parameters that were studied, and the use of DoE further saved time by minimizing the number of runs that were needed to understand that relationship between the process parameters and the CQAs. To the best of our knowledge, this is the first report of a combination risk analysis, DoE, MLR, and logistic regression approach applied to a microemulsion formulation. This combination of experimental design and modeling techniques was powerful, because it allowed us to use microemulsion composition to predict diameter, PDI, 30-day percent diameter change, and probability of meeting multiple CQA specifications. The QbD approach presented here can be adapted to other nanoformulations’ development and has the potential to reduce expenses through the prediction of unstable and/or unsuitable formulations in these early developmental stages. 

## 4. Materials and Methods 

### 4.1. Materials

Miglyol 812N was purchased from Fisher Scientific (Fair Lawn, NJ, USA). Kolliphor^®^ EL was purchased from Sigma-Aldrich (St. Louis, MO, USA). Polyethylene glycol (PEG) 400 monostearate, transcutol (2-(2-Ethoxyethoxy)ethanol), and propylene glycol were purchased from Spectrum Chemicals (CA, USA). Millex-GS syringe filters were purchased from EMD Millipore (Burlington, MA, USA). 

### 4.2. Microemulsion Production

Microemulsions were produced on a 100 mL scale via water titration. All microemulsion components (except water) were added to the beaker, with kolliphor EL and PEG 400 always added at a 1:1 *w*/*w* ratio, so that their total weight was equal to the specified surfactants weight. Excipients were stirred at a speed of 350 rpm for 30 min. Then, the stir rate was adjusted to 350 rpm or 700 rpm, and water titration was performed at the specified rate (4 mL/min or 12 mL/min). When water addition was complete, the microemulsion continued to be stirred for 60 min. All microemulsions were produced at ambient temperature, which varied between 18 and 22 °C.

### 4.3. Dynamic Light Scattering Measurements 

All dynamic light scattering measurements were performed using a Zetasizer Nano ZS series (Malvern Instruments, Worcestershire, UK). Microemulsions were diluted 1:40 *v*/*v* in de-ionized water. Measurements were performed at a temperature of 25 °C and a light scattering angle of 173°.

### 4.4. CQA Specification Testing

Filtration: After production, microemulsions were stored at ambient temperature. On day 1 (24 h) after production, 25 mL microemulsion were filtered through a Millex-GS syringe filter with a pore size of 0.22 µm and stored in a non-sterile, 50 mL plastic centrifuge tube. Diameter and polydispersity index (PDI) of filtered and unfiltered microemulsions were measured. Filtered microemulsion was stored at 4 °C, and unfiltered microemulsion was stored at ambient temperature. Centrifugation: Five days (120 h) after production, filtered microemulsions were diluted 1:40 *v*/*v* in de-ionized water and centrifuged at 1620× *g* for 30 min. Microemulsion diameter and PDI were then measured without additional sample dilution. Same day measurements of filtered microemulsion stored at 4 °C were used for comparison. Thermal Cycling: Immediately after filtration, 5 mL of undiluted, filtered microemulsion was added to a glass vial, sealed with parafilm, and stored at 4 °C. After 24 h, samples were moved to 50 °C. Every 24 h, vials were moved between 4 and 50 °C for a total of four thermal cycles (8 days). Upon completion, diameter and PDI of thermal cycling samples were measured after 1 h equilibration to room temperature. Same-day measurements of filtered microemulsion continuously stored at 4 °C were used for comparison.

### 4.5. Design of Experiments 

Screening Design: A two-level, seven factor, D-optimal screening mixture process variable design of experiments was developed using JMP Pro 13 software. The following constraints were defined for the mixture variables: Miglyol (2.0, 6.0% weight), transcutol (2.5, 7.5% weight), propylene glycol (0, 2.0% weight), and surfactants (Kolliphor EL and PEG 400, 1:1 *w*/*w*, 22.5, 27.5% combined weight). An additional constraint specified that the sum of Miglyol and propylene glycol could not exceed 6% weight for any formulation. Levels of 350 and 700 rpm and 4 and 12 mL/min were defined for stir rate and water addition rate, respectively. Factor ranges were selected based upon our prior knowledge in the field in combination with previous reports which studied the same parameters [14,15,16]. 

Augmented Design: Based upon statistical analysis of the screening DoE, this DoE was augmented to refine the design space and facilitate investigation of composition main effects and second order interactions. The augmented design consisted of the 15 screening runs plus 15 additional runs and modified the upper limit of internal phase such that it could not exceed 12% total weight. Additionally, the upper limit of propylene glycol was increased to 5% weight to capitalize on the finding that propylene glycol did not contribute significantly to studied CQA specifications. The selected augmented design maximized the power to detect regression coefficients.

### 4.6. Multiple Linear Regression (MLR) Modeling

JMP Pro 13 software was used to develop MLR models that predicted day 1 diameter, day 1 PDI, and 30-day percent diameter increase as a function of modeling process parameters. For screening studies, runs 1–15 (Table 4) were used to develop models that studied main effects terms only (five composition parameters, stir rate, and water addition rate). Models contained all seven parameters and were used solely to identify parameters that were likely to significantly impact (*p*-value < 0.05) the CQA specifications of interest. 

After screening studies, stir rate and water addition rate were determined to not significantly impact microemulsion CQA specifications (Table 7), and the design of experiments was augmented to include an additional 15 runs (runs 16–30, Table 4). This augmented, 30-run DoE was used to develop MLR models that predicted day 1 diameter, day 1 PDI, and 30-day percent diameter change as a function of microemulsion composition. All main effects and interactions were studied using a stepwise forward approach. All terms with a *p*-value < 0.05 were included in the models. Models were developed using 21 (75%) runs and validated using the remaining 7 (25%) runs. Validation sets were selected using a stratified random sampling of the output of interest. All models were developed without intercept terms. 

In all MLR models, mixture terms were coded as L pseudocomponents. This transformation is ${x\prime }_{i}=\frac{x_{i}-L_{i}}{\left(\mathrm{Total}-L\right)}\;$ where x’_i_ is the i’th pseudocomponent, x_i_ is the original component value, L_i_ is the lower constraint for the i’th component, L is the sum of lower constraints for all components, and Total is the mixture total. This linear transformation allows the regression coefficients for the mixture components to be comparable in size. 

### 4.7. Logistic Regression Modeling 

Logistic regression models were developed to predict the probability that a microemulsion would meet one or more CQA specifications. Microemulsions that met the CQA specification(s) were assigned a value of 1, and microemulsions that failed to meet one or both CQA specifications were assigned a value of 0. Models were developed using 21 (75%) runs and validated using the remaining 7 (25%) runs. The validation set was selected using a stratified random sampling of day 1 diameter (the predictor variable). 

### 4.8. Microemulsion Scale Up to 1 L

A selected microemulsion formulation (see results section for selection explanation) was scaled from 100 mL to 1000 mL in triplicate. Scaled up microemulsions were produced using a stir rate of 500 rpm and a water addition rate of 80 mL/min. Scaled up formulations underwent the same CQA specification testing as the 100 mL scale microemulsions. 

## 5. Conclusions

In the present work, we used quality by design methodology to efficiently identify stable, robust microemulsions and understand the processes that impact microemulsion diameter, PDI, and stability. We used FMECA to identify the process parameters that were most likely to impact microemulsion diameter, and through a screening design of experiments, we were able to identify a design space in which microemulsion diameter, PDI, and stability were dependent solely upon microemulsion composition. We hypothesized that microemulsions that were robust to changes in production processing parameters (stir rate and water addition rate) could undergo successful scale up. This hypothesis was confirmed, as we successfully and consistently scaled up a selected microemulsion tenfold, from 100 mL to 1000 mL. Using MLR, we were able to develop predictive models for microemulsion diameter, PDI, and 30-day percent diameter change. We also developed logistic regression models that predicted the probability that a microemulsion would meet one or more CQA specifications as a function of day 1 diameter. This unique combination of MLR and logistic regression was powerful in this specific application, as it could be used to predict not just the basic colloidal properties (diameter and PDI), but also the probability that a formulation will pass quality control testing. The present work is an example meant to demonstrate the usefulness of adapting QbD approaches to nanoformulation development. Adapting QbD approaches to nanoformulations has the potential to reduce expenses through early identification of unsuitable formulations, as well as increase the likelihood that the product can be scaled up for further study.

## Figures and Tables

**Table 1:** Critical quality attributes (CQAs), specifications, and brief descriptions of how CQAs are measured. All diameter and PDI measurements were performed with dynamic light scattering.

CQA	Specification	Brief Test Description
Microemulsion Diameter	<50 nm	Measured 24 h after production
Polydispersity Index (PDI)	<0.1	
Diameter Change After Filtration	<10%	0.22 μm mixed cellulose esters membrane
PDI After Filtration	<0.1	
Diameter Change After Centrifugation	<10%	1620× *g* for 30 min
PDI After Centrifugation	<0.1	
Diameter Change After Thermal Cycling	<10%	Moved between 4 °C and 50 °C every 24 h for 8 days
PDI After Thermal Cycling	<0.1	
30-day Diameter Change	<10%	Stored at ambient temperature
Day 30 PDI	<0.1	

**Table 2:** Descriptions for upper, middle, and lower risk priority number values for the categories of severity, frequency of occurrence, and detectability.

	1	3	5
Severity (S)	No appreciable consequence to batch quality	Requires action, but batch is recoverable	Total batch loss
Frequency of Occurrence (O)	Has not happened	Happens sporadically	Happens with regularity
Detectability (D)	Readily detected	Detected, but not always, or not in a timely manner	Not detectable within current manufacturing operation

**Table 3:** Abridged risk analysis with rankings. Using ranked risk priority numbers (RPNs), a methodical risk assessment identified the most influential parameters on microemulsion critical quality attributes. These parameters were studied in a screening mixture process variable design of experiments.

S	O	D	RPN	Method of Failure	CQA Impacted	Cause of Failure
5	3	5	75	Microemulsion diameter too large (>50 nm) and/or microemulsion PDI too large (>0.1)	Microemulsion diameter and/or microemulsion PDI	Water addition rate too fast
5	3	5	75			Stir rate too slow
3	1	5	15			Stirring time too short
5	1	5	25			Fluctuating room temperature
5	1	5	25			Vessel size incompatible with microemulsion volume
5	3	5	75			Oil:surfactant ratio too high
5	2	5	50			Incompatible excipients lead to phase separation
5	1	5	25	Diameter change too large (>10%) and/or PDI too large (>0.1)	Diameter change after filtration	Shear forces during filtration
5	2	5	50		Diameter increase and/or PDI after centrifugation	High oil:surfactant ratio causes droplet aggregation upon exposure to centrifugal force
5	4	5	100		Diameter increase and/or PDI after thermal cycling	High oil:surfactant ratio leads to droplet coalescence
5	2	5	50			High oil:surfactant ratio leads to phase separation
5	4	5	100		30-day diameter change or 30-day PDI	High oil:surfactant ratio leads to droplet coalescence
5	2	5	50			High oil:surfactant ratio leads to phase separation

**Table 4:** Runs in the design of experiments.

Run	Oil (% *w*/*v*)	Surfactants (% *w*/*v*)	Transcutol (% *w*/*v*)	Propylene Glycol (% *w*/*v*)	Water (% *w*/*v*)	Stir Rate (rpm)	Water Addition Rate (mL/min)
1	6	27.5	7.5	0	59	350	12
2 **	6	27.5	2.5	0	64	350	4
3 **	2	22.5	2.5	2	71	350	12
4	4	27.5	2.5	2	64	700	12
5	6	22.5	7.5	0	64	350	4
6	2	22.5	7.5	2	66	700	4
7	2	22.5	2.5	0	73	350	12
8 **	4	27.5	7.5	2	59	700	12
9	2	27.5	7.5	0	63	700	4
10	6	22.5	7.5	0	64	700	12
11	2	22.5	7.5	2	66	350	4
12	2	27.5	2.5	0	68	700	4
13 **	6	22.5	2.5	0	69	700	4
14	2	27.5	7.5	0	63	350	12
15	4	27.5	2.5	2	64	350	4
16	6	22.5	2.5	3.5	65.5	350	4
17	2	25	2.5	5	65.5	350	4
18	6	27.5	2.5	3.5	60.5	350	4
19	2	25.32	7.5	2.5	62.68	350	4
20	2	22.5	2.5	5	68	350	4
21	4.5	22.5	2.5	5	65.5	350	4
22	2	27.5	5	5	60.5	350	4
23	2	25.5	2.5	0	70	350	4
24	6	25	4.25	1.75	63	350	4
25	2	22.5	5	5	65.5	350	4
26	2	22.5	5.5	0	70	350	4
27	2	27.5	4.62	2	63.88	350	4
28	4.5	27.5	2.5	5	60.5	350	4
29	4	24.74	5.24	0	66.02	350	4
30	2	27.5	2.5	5	63	350	4

Runs 1–15 were part of a screening design that was used to identify parameters that significantly contributed to microemulsion diameter, PDI, and 30-day percent diameter increase. The design was then augmented to include runs 16–30 that enabled the study of interactions between these significant parameters. ** Indicates that the run was replicated in triplicate.

**Table 5:** Summary of CQA specification testing.

	CQA (Specification)									
Run	Diameter (<50nm)	PDI (<0.1)	Filtration Diameter Change (<10%)	Filtration PDI (<0.1)	Centrifugation Diameter Change (<10%)	Centrifugation PDI (<0.1)	Thermal Cycling Diameter Change (<10%)	Thermal Cycling PDI (<0.1)	30-day Diameter Change (<10%)	30-day PDI (<0.1)
1	33.56	0.087	1.39	0.086	1.39	0.106	74.58	0.071	48.88	0.240
2	29.25	0.055	0.87	0.052	0.87	0.072	28.49	0.079	16.83	0.114
3 *	18.40	0.046	−0.18	0.040	-0.18	0.029	1.71	0.024	2.68	0.040
4	21.51	0.052	0.57	0.042	0.57	0.039	17.04	0.102	10.33	0.095
5	116.50	0.110	0.09	0.118	0.09	0.082	−13.38	0.139	25.78	0.123
6 *	18.08	0.048	−0.20	0.028	−0.20	0.041	0.18	0.026	6.75	0.060
7 *	17.96	0.039	0.89	0.020	0.89	0.027	0.66	0.027	3.49	0.029
8	22.67	0.054	−0.38	0.049	−0.38	0.062	220.70	0.209	18.54	0.091
9 *	17.34	0.031	−0.67	0.031	−0.67	0.034	0.92	0.048	1.90	0.045
10	66.49	0.123	0.11	0.129	0.11	0.117	−5.51	0.089	42.11	0.119
11 *	18.32	0.022	−0.31	0.029	−0.31	0.045	0.63	0.035	5.82	0.049
12 *	17.06	0.037	1.19	0.026	1.19	0.019	−0.06	0.022	3.95	0.022
13	37.64	0.077	−0.20	0.085	−0.20	0.080	−1.77	0.070	31.09	0.115
14 *	17.27	0.029	0.02	0.035	0.02	0.060	0.15	0.029	2.10	0.036
15	22.23	0.054	−0.60	0.033	−0.60	0.044	14.22	0.108	11.35	0.154
16	50.62	0.106	−0.01	0.114	0.32	0.106	−4.70	0.089	30.20	0.103
17 *	17.57	0.059	−1.18	0.016	0.51	0.032	2.07	0.022	1.12	0.014
18	31.26	0.068	−0.29	0.073	0.09	0.098	30.74	0.065	17.32	0.122
19 *	17.20	0.033	−0.29	0.032	−0.56	0.036	−0.19	0.027	2.25	0.041
20 *	17.99	0.037	−2.00	0.030	0.39	0.029	1.22	0.026	3.48	0.026
21	29.90	0.085	0.08	0.069	−0.04	0.085	12.76	0.090	19.31	0.105
22 *	16.89	0.029	−0.53	0.044	−0.83	0.036	−0.24	0.022	1.03	0.028
23 *	17.21	0.035	0.35	0.043	0.00	0.037	1.04	0.018	2.79	0.035
24	39.40	0.103	−0.33	0.106	−0.15	0.103	−0.76	0.071	31.78	0.119
25 *	17.86	0.033	−1.06	0.037	−0.59	0.048	0.11	0.037	3.34	0.046
26 *	17.78	0.041	0.79	0.052	1.53	0.039	0.93	0.030	2.42	0.076
27 *	17.26	0.050	0.00	0.053	1.40	0.032	−0.45	0.032	−1.04	0.025
28	24.14	0.053	0.99	0.064	2.15	0.049	66.83	0.151	8.84	0.090
29	23.45	0.057	−1.46	0.048	−0.29	0.058	51.44	0.120	16.46	0.105
30 *	17.07	0.033	0.16	0.028	0.84	0.025	0.59	0.027	2.30	0.029

Values that meet the CQA specification are highlighted in gray. * Indicates the run met all CQA specifications.

**Table 6:** Four runs from the screening design of experiments were replicated in triplicate. All CQAs were measured for each replicate.

	Average ± SD			
	Run 2	Run 3	Run 8	Run 13
Diameter (nm)	28.87 ± 0.70	18.17 ± 0.33	22.66 ± 0.38	37.65 ± 0.56
PDI	0.0561 ± 0.0037	0.0337 ± 0.0146	0.0502 ± 0.0051	0.0767 ± 0.0003
Filtration Diameter (nm)	29.24 ± 0.63	18.11 ± 0.35	22.46 ± 0.51	37.43 ± 0.61
Filtration PDI	0.0496 ± 0.0030	0.0296 ± 0.0093	0.0462 ± 0.0057	0.0817 ± 0.0055
Centrifugation Diameter (nm)	30.78 ± 0.49	18.30 ± 0.36	23.32 ± 0.50	39.11 ± 0.14
Centrifugation PDI	0.0613 ± 0.0095	0.0289 ± 0.0004	0.0608 ± 0.0015	0.0810 ± 0.0030
Thermal Cycling Diameter (nm)	39.11 ± 1.37	18.52 ± 0.12	77.70 ± 7.46	41.80 ± 1.79
Thermal Cycling PDI	0.0737 ± 0.0044	0.0297 ± 0.0053	0.2304 ± 0.0547	0.0644 ± 0.0072
30-day Diameter (nm)	33.11 ± 1.02	18.45 ± 0.43	26.51 ± 0.89	47.43 ± 1.66
30-day PDI	0.1067 ± 0.0121	0.0366 ± 0.0051	0.1290 ± 0.0610	0.1176 ± 0.0056

**Table 7:** Left: Parameters and their corresponding *p*-values for MLR models developed from the screening design of experiments. Significant (*p*-value < 0.05) parameters are highlighted in gray. Right: Parameters and their corresponding percent contributions for boosted tree models developed from the screening design of experiments.

	MLR	
	Term	*p*-Value
Day 1 Diameter	(Oil − 0.02)/0.14	0.00586
	(Transcutol − 0.025)/0.14	0.04627
	(Water − 0.59)/0.14	0.14028
	(Surfactants − 0.225)/0.14	0.29011
	Water Addition Rate	0.61893
	Propylene Glycol/0.14	0.64347
	Stir Rate	0.65578
Day 1 PDI	(Oil − 0.02)/0.14	0.00002
	(Transcutol − 0.025)/0.14	0.00165
	(Water − 0.59)/0.14	0.00485
	(Surfactants − 0.225)/0.14	0.22251
	Water Addition Rate	0.25789
	Stir Rate	0.42514
	Propylene Glycol/0.14	0.43751
30 Day Diameter Change (%)	(Oil − 0.02)/0.14	0.00008
	(Transcutol − 0.025)/0.14	0.05528
	Water Addition Rate	0.17303
	(Surfactants − 0.225)/0.14	0.53077
	Stir Rate	0.56218
	(Water − 0.59)/0.14	0.60861
	Propylene Glycol/0.14	0.67400

**Table 8:** *R*^2^ and RASE for the MLR models developed from the augmented design of experiments. Training and validation sets consisted of 75% and 25% of the data, respectively. Validation sets were selected using a stratified random sampling of the output of interest.

CQA	Source	*R* ^2^	RASE
Day 1 Diameter (nm)	Training Set	0.9419	2.04 nm
	Validation Set	0.9908	0.81 nm
Day 1 PDI	Training Set	0.8949	0.0085
	Validation Set	0.8207	0.0087
30-day % Diameter Change	Training Set	0.9637	2.21%
	Validation Set	0.9878	1.41%

**Table 9:** Parameter estimates, standard errors, and *p*-values for the MLR models developed from the augmented design of experiments.

	Term	Estimate	Std Error	*p*-Value
Day 1 Diameter	(Oil − 0.02)/0.19	231.31	44.26	0.00013
	Propylene Glycol/0.19	15.20	5.32	0.01272
	(Transcutol − 0.025)/0.19	14.30	5.53	0.02149
	(Surfactants − 0.225)/0.19	15.85	5.76	0.01564
	(Water − 0.54)/0.19	18.29	1.62	<0.00001
	Oil × Surfactants	−367.22	84.41	0.00067
	Oil*Water	−139.94	63.81	0.04569
Day 1 PDI	(Oil − 0.02)/0.14	0.4955	0.0701	<0.00001
	(Transcutol − 0.025)/0.14	0.0349	0.0141	0.02545
	(Surfactants − 0.225)/0.14	0.0438	0.0142	0.00739
	(Water − 0.59)/0.14	0.0318	0.0055	0.00004
	Oil × Surfactants	−0.7934	0.1648	0.00023
	Oil × Water	−0.3178	0.1448	0.04435
30 Day % Diameter Change	(Oil − 0.02)/0.14	92.86	7.92	<0.00001
	(Water − 0.59)/0.14	4.87	1.27	0.00131
	Oil × Transcutol	291.59	27.58	<0.00001
	Oil × Surfactants	−106.37	26.87	0.00102

**Table 10:** Confusion tables for the logistic models that use day 1 microemulsion diameter to predict whether a microemulsion will meet the CQA specifications of thermal cycling diameter change and 30-day diameter change.

30-Day % Diameter Change				30-Day % Diameter Change and Thermal Cycling % Diameter Change			
Training		Predicted Count		Training		Predicted Count	
		1	0			1	0
Actual Count	1	12	1	Actual Count	1	12	0
	0	0	8		0	0	9
Validation		Predicted Count		Validation		Predicted Count	
		1	0			1	0
Actual Count	1	4	0	Actual Count	1	4	0
	0	1	2		0	0	3
**Day 30 PDI**				**Day 30 PDI and Thermal Cycling PDI**			
Training		Predicted Count		Training		Predicted Count	
		1	0			1	0
Actual Count	1	12	1	Actual Count	1	12	0
	0	0	8		0	0	9
Validation		Predicted Count		Validation		Predicted Count	
		1	0			1	0
Actual Count	1	5	0	Actual Count	1	4	0
	0	0	2		0	0	3

**Table 11:** Summary of CQA specification testing for scaled up (1000 mL) microemulsions.

	100 mL Scale (*n* = 1) AV	1000 mL Scale (*n* = 3) AV ± SD
Diameter (nm)	18.08	17.93 ± 0.19
PDI	0.0477	0.0444 ± 0.0165
Filtration Diameter (nm)	18.05	18.15 ± 0.50
Filtration PDI	0.0283	0.0449 ± 0.0170
Centrifugation Diameter (nm)	18.56	18.30 ± 0.48
Centrifugation PDI	0.0407	0.0382 ± 0.0107
Thermal Cycling Diameter (nm)	18.47	18.17 ± 0.05
Thermal Cycling PDI	0.0257	0.0354 ± 0.0049
30-Day Diameter (nm)	19.30	18.69 ± 0.19
30-Day PDI	0.0597	0.0561 ± 0.0096

The average and standard deviation of three scaled up microemulsions was calculated for each CQA test and compared to the result for the same formulation produced on a 100 mL scale.

## Supplementary Materials

The Supplementary Materials are available online.
